# Updated systematic review and network meta-analysis of first-line treatments for metastatic renal cell carcinoma with extended follow-up data

**DOI:** 10.1007/s00262-023-03621-1

**Published:** 2024-01-30

**Authors:** Takafumi Yanagisawa, Keiichiro Mori, Akihiro Matsukawa, Tatsushi Kawada, Satoshi Katayama, Kensuke Bekku, Ekaterina Laukhtina, Pawel Rajwa, Fahad Quhal, Benjamin Pradere, Wataru Fukuokaya, Kosuke Iwatani, Masaya Murakami, Karim Bensalah, Viktor Grünwald, Manuela Schmidinger, Shahrokh F. Shariat, Takahiro Kimura

**Affiliations:** 1 Department of Urology, Comprehensive Cancer Center, Medical University of Vienna, Wahringer Gurtel 18-20, 1090 Vienna, Austria; 2 Department of Urology, The Jikei University School of Medicine, Tokyo, Japan; 3 Department of Urology, Dentistry and Pharmaceutical Sciences, Okayama University Graduate School of Medicine, Okayama, Japan; 4 Institute for Urology and Reproductive Health, Sechenov University, Moscow, Russia; 5 Department of Urology, Medical University of Silesia, Zabrze, Poland; 6 Department of Urology, King Fahad Specialist Hospital, Dammam, Saudi Arabia; 7 Department of Urology, La Croix Du Sud Hospital, Quint Fonsegrives, France; 8 Department of Urology, University of Rennes, Rennes, France; 9 Clinic for Medical Oncology and Clinic for Urology, West German Cancer Center Essen, University Hospital Essen, Essen, Germany; 10 Division of Urology, Department of Special Surgery, The University of Jordan, Amman, Jordan; 11 Department of Urology, University of Texas Southwestern Medical Center, Dallas, TX USA; 12 Department of Urology, Second Faculty of Medicine, Charles University, Prague, Czech Republic; 13 Department of Urology, Weill Cornell Medical College, New York, NY USA; 14 Karl Landsteiner Institute of Urology and Andrology, Vienna, Austria

**Keywords:** Immune checkpoint inhibitors, Renal cell carcinoma, Metastasis, Network meta-analysis

## Abstract

Immune checkpoint inhibitor (ICI)-based combination therapies are the recommended first-line treatment for metastatic renal cell carcinoma (mRCC). However, no head-to-head phase-3 randomized controlled trials (RCTs) have compared the efficacy of different ICI-based combination therapies. Here, we compared the efficacy of various first-line ICI-based combination therapies in patients with mRCC using updated survival data from phase-3 RCTs. Three databases were searched in June 2023 for RCTs that analyzed oncologic outcomes in mRCC patients treated with ICI-based combination therapies as first-line treatment. A network meta-analysis compared outcomes including overall survival (OS), progression-free survival (PFS), objective response rate (ORR), and complete response (CR) rate. Subgroup analyses were based on the International mRCC Database Consortium risk classification. The treatment ranking analysis of the entire cohort showed that nivolumab + cabozantinib (81%) had the highest likelihood of improving OS, followed by nivolumab + ipilimumab (75%); pembrolizumab + lenvatinib had the highest likelihood of improving PFS (99%), ORR (97%), and CR (86%). These results remained valid even when the analysis was limited to patients with intermediate/poor risk, except that nivolumab + ipilimumab had the highest likelihood of achieving CR (100%). Further, OS benefits of ICI doublets were not inferior to those of ICI + tyrosine kinase inhibitor combinations. Recommendation of combination therapies with ICIs and/or tyrosine kinase inhibitors based on survival benefits and patient pretreatment risk classification will help advance personalized medicine for mRCC.

**Supplementary Information:**

The online version contains supplementary material available at 10.1007/s00262-023-03621-1.

## Introduction

The treatment of metastatic renal cell carcinoma (mRCC) has changed considerably with the development of immune checkpoint inhibitors (ICIs) [1, 2]. To date, five different ICI-based systemic combination therapies, including ICI + ICI or ICI + tyrosine kinase inhibitor (TKI), have been recommended as first-line treatment options for mRCC based on the International mRCC Database Consortium (IMDC) risk classification [1]. However, no head-to-head phase 3 randomized controlled trials (RCTs) have compared the efficacy of different ICI-based combination therapies, making optimal treatment selection difficult. Several network meta-analyses (NMAs) have investigated the efficacy and safety profiles of these combination therapies, suggesting that pembrolizumab + lenvatinib provides the greatest overall survival (OS) benefit [3–5].

However, heterogeneity in patient populations (i.e., different proportions of patients in the IMDC risk categories) and insufficient follow-up have made OS comparisons unreliable. Recently, the survival data of some of these RCTs were updated with additional follow-up data [6–9]. Therefore, this study present updated an NMA using this updated survival data to compare the efficacy of first-line ICI-based combination therapies in patients with mRCC, stratified by IMDC risk classification.

## Methods

The protocol of this study has been registered in the International Prospective Register of Systematic Reviews database (PROSPERO: CRD42023440048).

### Search strategy

This systematic review and NMA was conducted based on the guidelines of the Preferred Reporting Items for Systematic Reviews and Meta-Analyses (PRISMA) statement and PRISMA for NMA (Supplementary Table 1) [10, 11]. PubMed®, Web of Science™, and Scopus® databases were searched in June 2023 to identify studies investigating oncologic outcomes in mRCC patients treated with ICI-based combination therapies as a first-line treatment. The detailed search words were listed in Supplementary Fig. 7 and Supplementary Appendix 1. Subsequently, we reviewed abstracts from recent major conferences, such as the American Society of Clinical Oncology and the European Society for Medical Oncology, to include trial updates. The outcome measures of interest were OS, progression-free survival (PFS), objective response rates (ORRs), complete response (CR) rates, and treatment related adverse events (TRAEs).

The titles and abstracts were independently screened by two investigators. Potentially relevant studies were subjected to full-text review. Disagreements were resolved by establishing consensus among co-authors.

### Inclusion and exclusion criteria

Studies were included if they investigated patients with mRCC (Participants) and compared the efficacy of guideline-recommended ICI-based combination therapies (Interventions) with the efficacy of standard of care at the time of study enrollment (Comparisons) to assess their differential effects on OS, PFS, ORRs, CR rates, and/or TRAEs (Outcome) in RCTs (Study design). Studies lacking original patient data, reviews, letters, editorial comments, replies from authors, case reports, and articles not written in English were excluded. Relevant references of eligible studies were scanned for additional studies of interest.

### Data extraction

Two authors independently extracted the relevant data as follows: studies and the first author’s name; publication year; inclusion criteria; agents, dosage, and control arms; median age; number of patients stratified by IMDC risk classification; follow-up periods; TRAE, ORRs; CR rates; and duration of response rates. Hazard ratios (HRs) and 95% confidence intervals (CIs) from Cox regression models for OS and PFS were extracted. All discrepancies were resolved by establishing consensus among the co-authors of this study. As the CLEAR trial failed to show the superiority of everolimus + lenvatinib over sunitinib alone, only data on pembrolizumab + lenvatinib versus sunitinib were extracted [12].

### Risk of bias assessment

We evaluated the quality and risk of bias of eligible RCTs according to the Cochrane Handbook for Systematic Reviews of Interventions risk-of-bias tool (RoB version 2) (Supplementary Fig. 1) [13]. The risk-of-bias assessment of each study was independently performed by two authors.

### Statistical analyses

All eligible RCTs reported the oncologic and safety outcomes in overall population as well as patients stratified by IMDC risk classification (favorable and intermediate/poor risks). We conducted an NMA using random-effect models for direct and indirect treatment comparisons across outcomes [14, 15]. Contrast-based analyses were applied with estimated differences in the log HR and the standard error calculated from the HRs and CI [16]. The relative effects were presented as HRs or odds ratios (ORs) and 95% CI [14]. Different regimens were ranked in terms of OS, PFS, ORRs, CR rates, and TRAEs rates using the surface under the cumulative ranking (SUCRA) [14]. Additionally, we performed subgroup analyses for each outcome separately in patients with favorable or intermediate/poor risk. Network plots were created to illustrate the connectivity of the treatment networks. All statistical analyses were performed using R version 4.2.2 (R Foundation for Statistical Computing, Vienna, Austria).

## Results

### Study selection and characteristics

The PRISMA flow chart detailing our study selection process is shown in Supplementary Fig. 7. An initial literature search identified 8,548 records. After removing duplicates, 6,425 records remained for title and abstract screening. After screening, we performed a full-text review of 47 articles, leading to the final identification of 5 RCTs including 7 updates comprising 4,206 mRCC patients treated with ICI-based combination therapy [6–9, 12, 17–23]. The study and patient demographics of eligible RCTs are described in Table 1. All five RCTs provided data on differential OS, PFS, ORRs, and CR rates stratified by IMDC risk classification. Sunitinib alone, nivolumab + cabozantinib, nivolumab + ipilimumab, pembrolizumab + lenvatinib, pembrolizumab + axitinib, and avelumab + axitinib were included in this NMA. After updating the follow-up, median follow-up duration ranged from 33.6 to 67.7 months.

**Table 1:** Study demographics and oncologic outcomes of included RCTs of 1st-line ICI-based combination therapy for mRCC

Study name and first author	Year	Treatment arm	Control arm	No. of patients		Median F/U, mo	Oncologic outcomes in overall population											
							ORR (%)		CR (%)		DOR		PFS			OS		
											Median, mo		Median, mo		HR (95%CI)	Median, mo		HR (95%CI)
				T	C		T	C	T	C	T	C	T	C		T	C	
CheckMate9ER Motzer et al	2022 2023	Nivolumab + Cabozantinib	Sunitinib	323	328	44.0 (min: 36.5)	180 (55.7)	93 (28.4)	40 (12.4)	17 (5.2)	23.1	15.2	16.6	8.4	0.58 (0.48–0.71)	49.5	35.5	0.70 (0.56–0.87)
JAVELIN Renal 101 Motzer et al. Choueiri et al	2019 2022	Avelumab + Axitinib	Sunitinib	442	444	T: 34.1 C: 33.6	262 (59.3)	141 (31.8)	21 (4.8)	14 (3.2)	19.4	14.5	13.9	8.9	0.67 (0.568–0.785)	NR	37.8	0.79 (0.643–0.969)
KEYNOTE-426 Powles et al	2020 2023	Pembrolizumab + Axitinib	Sunitinib	432	429	67 (min: 60)	262 (60.6)	170 (39.6)	50 (11.6)	17 (4.0)	23.6	15.3	15.7	11.1	0.69 (0.59–0.81)	47.2	40.8	0.84 (0.71–0.99)
CheckMate214 Motzer et al	2018 2022	Nivolumab + Ipilimumab	Sunitinib	550	546	67.7	216 (39.3)	177 (32.4)	64 (11.6)	17 (3.1)	NR	24.8	12.3	12.3	0.86 (0.73–1.01)	55.7	38.4	0.72 (0.62–0.85)
CLEAR Motzer et al	2021 2023	Pembrolizumab + Lenvatinib	Sunitinib	355	357	T: 49.8 C: 49.4	253 (71.3)	131 (36.7)	65 (18.3)	17 (4.8)	26.7	14.7	23.9	9.2	0.47 (0.38–0.57)	53.7	54.3	0.79 (0.63–0.99)

*RCTs* Randomized controlled trials, *ICI* immune checkpoint inhibitors, *mRCC* metastatic renal cell carcinoma, *No.* number, *T* treatment arm, *C* control arm, *IMDC* international metastatic RCC database consortium, *F/U* follow-up, *mo.*: months, *ORR* objective response rate, *ITT* intention-to-treat, *DOR* duration of response, *OS* overall survival, *PFS* progression-free survival, *HR* hazard ratio, *CI* confidence interval, *NR* not reported

### Risk of bias assessment

All included phase 3 RCTs had a low risk of bias or some concerns (Supplementary Fig. 1). The quality assessment was conducted using the AMSTAR2 checklist; overall confidence in the results of this NMA was “High” (Supplementary Appendix 2) [24]

### Network meta-analysis of oncologic outcomes

Network plots for all oncologic outcomes were depicted in Supplementary Fig. 2. The results of treatment rankings are summarized in Table 2.

**Table 2:** Summary of results of treatment rankings based on SUCRA analysis among five different ICI-based combinations

Outcomes	Population	Nivolumab + Ipilimumab	Nivolumab + Cabozantinib	Pembrolizumab + Lenvatinib	Pembrolizumab + Axitinib	Avelumab + Axitinib	Sunitinib
OS	Overall	2	1	3	5	4	6
	Favorable risk	2	5	3	6	1	4
	Intermediate/Poor risk	2	1	3	4	5	6
PFS	Overall	5	2	1	4	3	6
	Favorable risk	6	3	1	4	2	5
	Intermediate/Poor risk	5	2	1	4	3	6
ORR	Overall	5	2	1	4	3	6
	Favorable risk	6	2	4	3	1	5
	Intermediate/Poor risk	5	2	1	4	3	6
CR	Overall	2	4	1	3	5	6
	Favorable risk	3	5	1	2	4	6
	Intermediate/Poor risk	1	4	2	3	5	6

*SUCRA* surface under the cumulative ranking, *ICI* immune checkpoint inhibitor, *OS* overall survival, *PFS* progression-free survival, *ORR* objective response rate, *CR* complete response

#### Overall population

##### OS and PFS

**Fig. 1:**
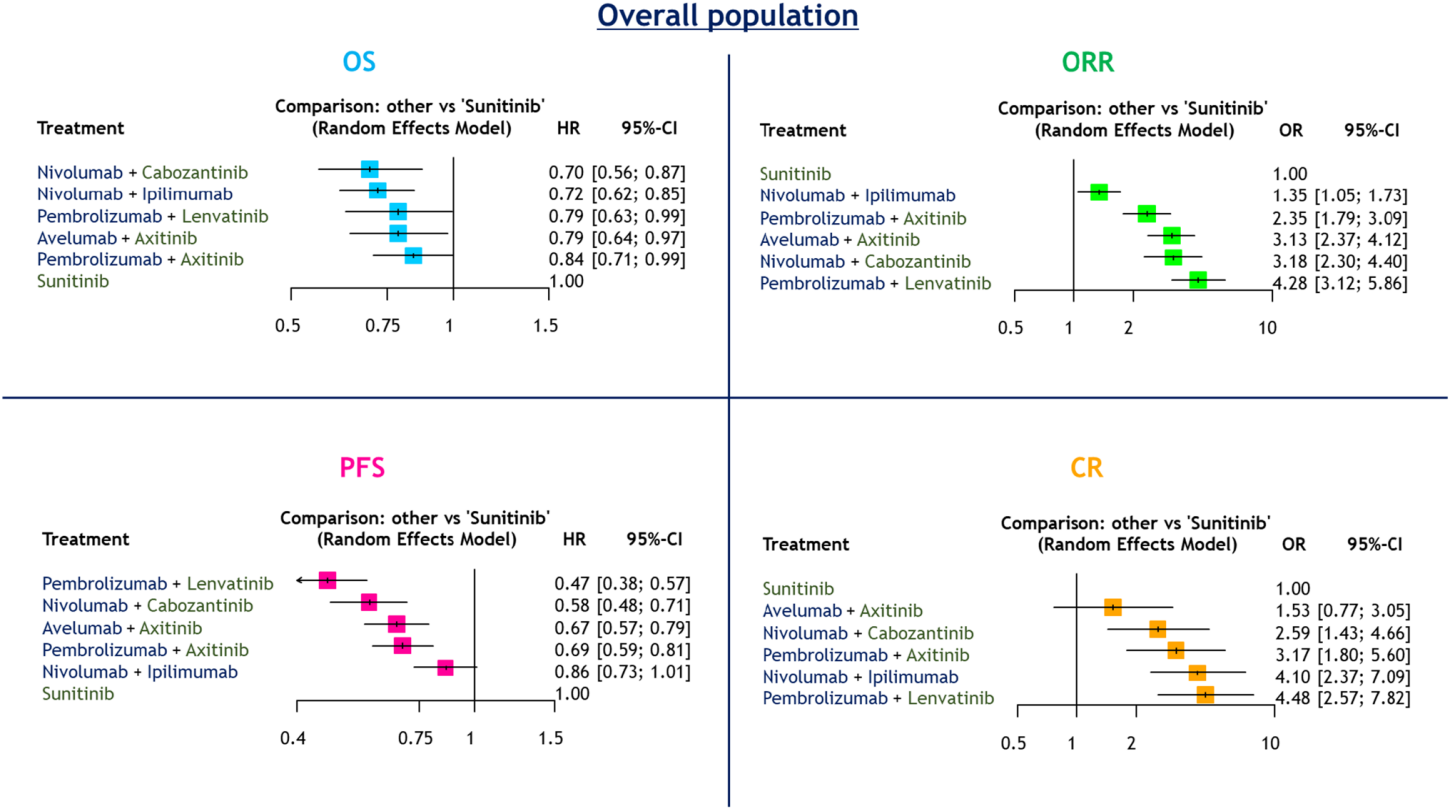
Forest plots showing the results of NMA among the overall population for OS, PFS, ORR, and CR in mRCC patients treated with first-line ICI-based combination therapy

Compared to sunitinib alone, all ICI-based combinations resulted in improved OS in patients with mRCC (Fig. 1). Treatment rankings based on the SUCRA analysis revealed that nivolumab + cabozantinib (81%) had the highest likelihood of providing the maximal OS benefit, followed by nivolumab + ipilimumab (75%), pembrolizumab + lenvatinib (54%), avelumab + axitinib (51%), pembrolizumab + axitinib (38%), and sunitinib (1.2%: Supplementary Fig. 3A).

Compared to sunitinib alone, all ICI-based combinations other than nivolumab + ipilimumab resulted in improved PFS. Treatment rankings revealed that pembrolizumab + lenvatinib (99%) had the highest likelihood of providing the maximal PFS benefit, followed by nivolumab + cabozantinib (77%), avelumab + axitinib (55%), pembrolizumab + axitinib (49%), nivolumab + ipilimumab (20%), and sunitinib (0.7%: Supplementary Fig. 3B).

##### ORRs and CR rates

Compared to sunitinib alone, all ICI-based combinations resulted in improved ORRs (Fig. 1). Treatment rankings revealed that pembrolizumab + lenvatinib (97%) had the highest likelihood of providing the maximal ORR benefit, followed by nivolumab + cabozantinib (72%), avelumab + axitinib (69%), pembrolizumab + axitinib (43%), nivolumab + ipilimumab (20%), and sunitinib (0%: Supplementary Fig. 3C).

All ICI-based combinations other than avelumab + axitinib resulted in improved CR rates compared to sunitinib alone (Fig. 1). Treatment rankings revealed that pembrolizumab + lenvatinib (86%) had the highest likelihood of providing the maximal CR benefit, followed by nivolumab + ipilimumab (80%), pembrolizumab + axitinib (61%), nivolumab + cabozantinib (48%), avelumab + axitinib (22%), and sunitinib (2.2%: Supplementary Fig. 3D).

##### TRAEs

Compared to sunitinib alone, only ipilimumab + nivolumab were associated with significantly more favorable TRAEs (Supplementary Fig. 4). Treatment rankings revealed that ipilimumab + nivolumab had the highest likelihood of providing the most favorable TRAE profile.

#### Patients with favorable risk

##### OS and PFS

Compared to sunitinib alone, all ICI-based combinations failed to show the OS benefit in mRCC patients with favorable risk (Supplementary Fig. 8). Treatment rankings based on the SUCRA analysis revealed that avelumab + axitinib (85%) had the highest likelihood of providing the maximal OS benefit, followed by nivolumab + ipilimumab (55%), pembrolizumab + lenvatinib (54%), sunitinib (44%), nivolumab + cabozantinib (34%), and pembrolizumab + axitinib (28%: Supplementary Fig. 5A).

Compared to sunitinib alone, only pembrolizumab + lenvatinib (HR: 0.50, 95%CI: 0.35–0.71) significantly improved PFS (Supplementary Fig. 8). Treatment rankings revealed that pembrolizumab + lenvatinib (96%) had the highest likelihood of providing the maximal PFS benefit, followed by avelumab + axitinib (64%), nivolumab + cabozantinib (62%), pembrolizumab + axitinib (55%), sunitinib (22%), and nivolumab + ipilimumab (0%: Supplementary Fig. 5B).

##### ORRs and CR rates

Compared to sunitinib alone, all ICI-based combinations other than nivolumab + ipilimumab resulted in improved ORRs (Supplementary Fig. 8). Treatment rankings revealed that avelumab + axitinib (92%) had the highest likelihood of providing the maximal ORR benefit, followed by nivolumab + cabozantinib (70%), pembrolizumab + axitinib (61%), pembrolizumab + lenvatinib (57%), sunitinib (20%), and nivolumab + ipilimumab (0%: Supplementary Fig. 5C).

Only pembrolizumab + lenvatinib (OR: 5.20, 95%CI: 2.03–13.31) combinations resulted in significantly improved CR compared to sunitinib alone (Supplementary Fig. 8). Treatment rankings revealed that pembrolizumab + lenvatinib (94%) had the highest likelihood of providing the maximal CR benefit, followed by pembrolizumab + axitinib (57%), nivolumab + ipilimumab (53%), avelumab + axitinib (47%), nivolumab + cabozantinib (43%), and sunitinib (6.6%: Supplementary Fig. 5D).

#### Patients with intermediate/poor risk

##### OS and PFS

**Fig. 2:**
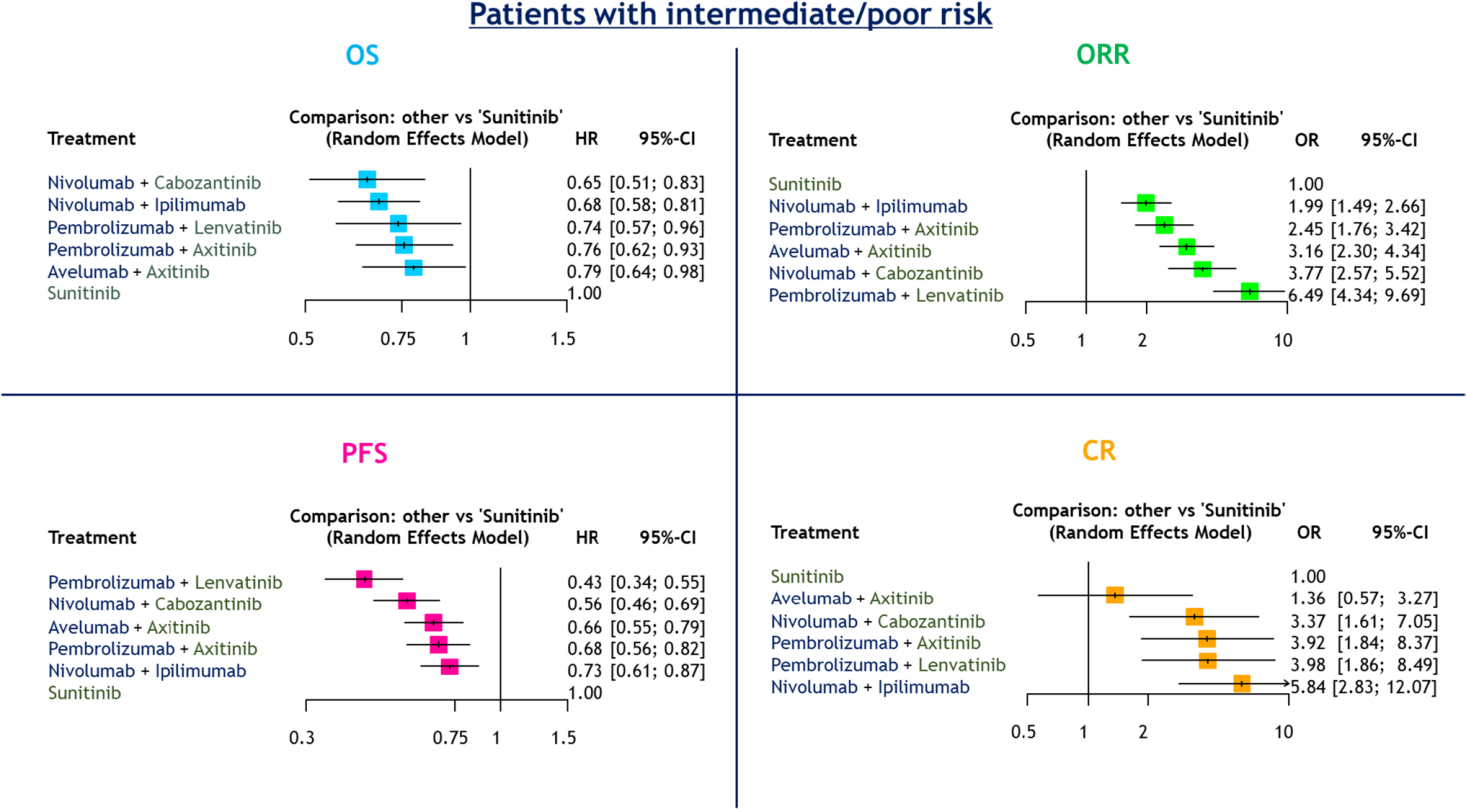
Forest plots showing the results of NMAs for OS, PFS, ORR, and CR in mRCC patients with intermediate/poor risk treated with first-line ICI-based combination therapy

Compared to sunitinib alone, all ICI-based combinations resulted in improved OS in mRCC patients with intermediate/poor risk (Fig. 2). Treatment rankings based on the SUCRA analysis revealed that nivolumab + cabozantinib (83%) had the highest likelihood of providing the maximal OS benefit, followed by nivolumab + ipilimumab (74%), pembrolizumab + lenvatinib (53%), pembrolizumab + axitinib (48%), and avelumab + axitinib (41%: Supplementary Fig. 6A).

Compared to sunitinib alone, all ICI-based combinations resulted in improved PFS (Fig. 2). Treatment rankings revealed that pembrolizumab + lenvatinib (99%) had the highest likelihood of providing the maximal PFS benefit, followed by nivolumab + cabozantinib (76%), avelumab + axitinib (49%), pembrolizumab + axitinib (44%), nivolumab + ipilimumab (31%), and sunitinib (0%: Supplementary Fig. 6B).

##### ORRs and CR rates

Compared to sunitinib alone, all ICI-based combinations resulted in improved ORRs (Fig. 2). Treatment rankings revealed that pembrolizumab + lenvatinib (100%) had the highest likelihood of providing the maximal ORR benefit, followed by nivolumab + cabozantinib (74%), avelumab + axitinib (62%), pembrolizumab + axitinib (40%), nivolumab + ipilimumab (24%), and sunitinib (0%: Supplementary Fig. 6C).

All ICI-based combinations other than avelumab + axitinib resulted in improved CR rates compared to sunitinib alone (Fig. 2). Treatment rankings revealed that nivolumab + ipilimumab (88%) had the highest likelihood of providing the maximal CR benefit, followed by pembrolizumab + lenvatinib (66%), pembrolizumab + axitinib (66%), nivolumab + cabozantinib (57%), avelumab + axitinib (17%), and sunitinib (5.3%: Supplementary Fig. 3).

## Discussion

At present, ICI-based combination therapies (ICI + ICI or ICI + TKI) are the major first line treatment for mRCC [1, 2]. However, the survival data, particularly OS data, available for IC + TKI is insufficient, rendering comparisons between the survival benefits of ICI + ICIs and ICI + TKI difficult [3–5]. Therefore, in our NMA, we compared these combination therapies based on recently reported long-term follow-up data and demonstrated several important findings. First, nivolumab + cabozantinib was associated with favorable OS outcomes during long-term follow-up. Second, pembrolizumab + lenvatinib had inferior OS benefits compared to nivolumab + cabozantinib or nivolumab + ipilimumab, despite being associated with extremely favorable PFS, ORR, and CR outcomes. Third, avelumab + axitinib was associated with superior OS and ORR, thus representing the best treatment option for patients at favorable risk. Fourth, nivolumab + ipilimumab was associated with the best CR rates and favorable OS outcomes among patients at intermediate/poor risk, despite having inferior ORR outcomes. Fifth, of the TRAEs evaluated for all regimens, not only any TRAEs but severe TRAEs were shown to be the most favorable with ipilimumab + nivolumab.

Based on recently reported long-term follow-up data, the Kaplan–Meier survival curves were reported to become increasingly less separate between pembrolizumab + axitinib or lenvatinib and the control treatments after approximately 3 years of follow-up, but remained distinct between nivolumab + cabozantinib and the control treatments. Therefore, nivolumab + cabozantinib is likely favorable over pembrolizumab-based therapies. ICI + TKI combinations have emerged as key treatment strategies for enhancing tumor responses and improving survival outcomes. TKIs can enhance the effectiveness of ICIs by affecting tumor microenvironments via their antiangiogenic effects, thereby increasing cytotoxic T-cell activity and infiltration [25]. ICIs are also believed to reciprocally enhance the benefits of TKIs [26]. Additionally, RCC is immunogenic and proangiogenic, and the immune system is believed to play a major role in promoting tumor resistance to TKIs in RCC [5, 27, 28].

In the context of TKI resistance, cabozantinib needs to be considered in combination with nivolumab-based therapy and has been associated with long-term efficacy in RCC. Unlike conventional TKIs, cabozantinib is a multi-TKI exhibiting broad-spectrum activity against VEGFR, GAS6, MET, AXL, MER, and TYRO3 [29, 30]. Notably, MET and AXL (both known to be involved in the survival, proliferation, infiltration, and metastasis of tumor cells as well as in the mechanisms of tumor resistance to molecularly targeted agents) were reported to be overexpressed in RCC. In addition, HGF, a MET ligand secreted mainly from mesenchymal cells in tumor tissues, exerts a wide array of physiological effects, including promoting tumor cell proliferation and inhibiting tumor cell apoptosis [31, 32]. GAS6, an AXL ligand expressed under serous fasting states resulting in tumor cell growth arrest, is involved in tumor metastasis and infiltration [33, 34]. Therefore, activation of the HGF-MET and GAS6-AXL pathways promotes tumor survival, proliferation, infiltration, and metastasis [35–37], and blocking VEGF can lead to MET and AXL activation.

Several reports have suggested that cabozantinib promotes a tumor microenvironment conducive to robust immune responses and is thus synergistic with ICIs. Cabozantinib inhibits HGF-induced PD-L1 expression in renal cancer cell-injected mouse models [38], indicating that it can prevent tumor cell immune escape through HGF/c-MET signaling. Moreover, the BAS6/AXL pathway is involved in the immunoinhibitory effects mediated by regulatory T (Tregs) or natural killer (NK) cells [39, 40], and the VEGFR pathway is involved in immunosuppression by promoting T-cell migration, inhibiting dendritic cell maturation, and promoting Treg and myeloid-derived suppressor cell (MDSC) maturation. These findings suggest that inhibition of AXL and VEGFR promotes antitumor immunity [41]. Notably, treatment with cabozantinib increases the expression of major histocompatibility complex (MHC) class I antigens in MC38-CEA mouse tumor cells and the number of peripheral CD8 + T-cells while decreasing the number of Tregs and MDSCs in a MC38CEA mouse colon cancer model [42]. Cabozantinib + ICI combination therapy is shown to have synergistic antitumor effects, resulting in reduced numbers of MDSCs alongside an increase in CD8 + T-cells and the ratio of CD8 + T-cells/Tregs in a mouse model of metastatic castration-resistant prostate cancer (mCRPC) [43]. Furthermore, a phase II trial in patients with metastatic, triple-negative breast cancer showed that cabozantinib continuously increased the number of circulating CD3 + T-lymphocytes while continuously decreasing CD14 + monocytes, suggesting that cabozantinib treatment led to bolstered antitumor immunity [44]. In summary, MET signaling is assumed to inhibit tumor immune responses by increasing PD-L1 expression, promoting the differentiation of T-cells into Tregs, increasing immunoinhibitory enzyme IDO-1 activity, and promoting the production of the immunosuppressive cytokine TGF-β [31, 32] AXL signaling is assumed to inhibit the antitumor activity of activated macrophages, dendritic cells, and NK cells [33, 34]. Therefore, cabozantinib therapy targets the tumor vasculature and tumor cells, inducing potent immunomodulatory effects that render it suitable for use in IC + TKI combination therapies [30].

However, these findings should be interpreted cautiously, particularly those on OS, as different TKI regimens and/or anti-PD-L1 antibodies were used. Moreover, the study populations varied among the studies, and subsequent treatment rates may have greatly affected the results. In interpreting the results reported herein, caution should be exercised to take into account factors that may have worked in favor of nivolumab + cabozantinib as well as in disfavor of pembrolizumab + lenvatinib, which, in turn, may account in part for the discordance between the OS and PFS/ORR outcomes with these regimens. Additionally, of note, patients treated with anti-PD-1/PD-L1 antibodies accounted for a greater proportion of the study populations in the KeyNote-26 (55.9%) and KeyNote-581 (54.6%) trials than in the CheckMate-9ER trial (31%). This may have positively affected those treated with sunitinib and decreased the difference in OS between those treated with pembrolizumab + lenvatinib or axitinib combinations and those treated with sunitinib alone. Furthermore, patients with favorable IMDC risk accounted for approximately 22% of the study population in the CheckMate-9ER trial, but > 30% of the study population in the KeyNote-426 and -581 trials, which may have affected the OS findings. Those with poor IMDC risk accounted for approximately 20% of the study population in the CheckMate-9ER trial but only 10% in the KeyNote-426 and -581 trials. In the KeyNote-581 trial, the HR for OS slightly favored sunitinib alone (HR, 0.85) over ICI + TKI combination therapy in a subgroup analysis of patients with an intermediate IMDC risk. Therefore, it is speculated that of all patients with intermediate-risk IMDC, more patients with a relatively favorable prognosis (who benefited more with sunitinib alone) were enrolled in the KeyNote-581 trial. Notably, the KeyNote-581 trial had more censored cases at 36 months, which coincides with the fact that the difference in the Kaplan–Meier survival curves began to diminish. In addition, among the patients treated with nivolumab + cabozantinib, approximately 7% and 8% discontinued treatment due to AEs associated with cabozantinib and nivolumab, respectively, indicating good overall tolerance [45]. In contrast, approximately 26% and 29% patients discontinued treatment due to adverse events associated with lenvatinib and pembrolizumab, respectively [45]. The study results may also have been affected by whether patients with RCC complied with their long-term treatments, as initial treatment with TKI + ICI may be effective.

Our risk-stratified analysis enabled us to characterize the efficacy of each treatment regimen and generate additional insights. We demonstrated that avelumab + axitinib was the best treatment option for patients with favorable IMDC risk and led to good OS and ORR outcomes. Meanwhile, nivolumab + ipilimumab produced the best CR rates among those at intermediate IMDC risk. Although many factors may have contributed to these results, the presence of angiogenic and immunogenic molecular subsets among patients with RCC is of special interest. The angiogenic and immunogenic subsets account for the majority and minority of those with favorable IMDC risk, respectively. In contrast, the immunogenic subset accounts for a greater proportion of those with poor IMDC risk than the angiogenic subset [46], suggesting that the best treatment option for RCC may vary depending on patients’ pretreatment risk. However, the paucity of study data available for analysis only allowed patients with intermediate/poor IMDC risk to be assessed in this study. This led to a heterogenous population requiring separate analysis as two distinct risk groups, and therefore caution is needed when interpreting our results. Additionally, avelumab + axitinib has not been recommended as a preferred regimen in major guidelines, given its failure to meet the primary endpoint in the JAVELIN Renal 101 study. Indeed, a comparison of OS Kaplan–Meier curves for favorable-risk patients in the four RCTs evaluated in this review shows that the OS curves begin to separate between the control (sunitinib) and the treatment (ICI + TKI) groups only 2 years after study initiation even in the JAVELIN Renal 101 study in which the treatment appeared to fare marginally better than the control. Again, the duration of ICI therapy was not restricted in the JAVELIN Renal 101 study but was limited to 2 years in the other three RCTs, suggesting that 2 years of ICI therapy may not be adequate and a longer duration of ICI therapy may be required in favorable-risk patients with favorable prognosis. In other words, the results from analysis of favorable-risk patients in this review may have primarily reflected differences in duration of ICI therapy among the RCTs compared. Thus, this limitation needs to be taken into account when interpreting the results of the present analysis and the results for favorable-risk patients should be deemed inconclusive and referred to only as a guide pending results of a final OS analysis becoming available from the JAVELIN Renal 101 study.

Despite its comprehensive nature, this study had several limitations. First, this NMA depended on the reporting quality and reliability of the reviewed trials, which may have suffered from bias, thus limiting the validity of its findings. Second, although the study used indirect treatment comparisons of RCT outcomes, it was not intended to replace head-to-head comparisons in clinical trials. Furthermore, given that the present analysis found it difficult to adequately adjust for these differences in patient characteristics among the RCTs evaluated, it should be noted that this may account in part for the discordance between the OS and PFS/ORR outcomes in its analysis of oncological outcomes. Third, CR rates vary largely depending on a prior history of nephrectomy; those not having undergone nephrectomy had larger tumor volumes, which likely contributed to decreased CR rates, and vice versa. Fourth, considering that some of the updated data included in this analysis remain to be published, this meta-analysis may have suffered from missing data. Fifth, while a brief analysis of TRAEs was performed for the treatment options evaluated, no detailed analysis of AEs was performed in this review primarily focused on their efficacy profiles. The caveat is therefore that in choosing among the ICI-based treatments, full consideration needs to be given not only to their respective oncological efficacy but to their respective safety profiles and potential AEs. Sixth, the COSMIC-313 trial was excluded from the present analysis because of the lack of OS data despite its favorable PFS and improved progressive disease rates/ORRs [47]. Moreover, the COSMIC-313 trial has also been associated with an increased incidence of AEs and low CR rates, thus raising concerns about whether PFS outcomes actually translate into improved OS. Therefore, long-term follow-up is required to obtain robust OS data for this RCT. Finally, considering that the RCTs evaluated in this study offered a limited range of effective options as second- or later-line treatment, and that ICI rechallenge may not be an option (in light of the negative results from the CONTACT trial), selection of an appropriate first-line treatment is critical [48, 49].

## Conclusions

The present analysis, based on updated follow-up data, revealed the varying efficacy of ICI combination therapies. Our updated NMAs revealed that the OS benefits of nivolumab + ipilimumab was not inferior to those of other ICI + TKI regimens. The outcomes of this regimen in patients with intermediate/poor IMDC risk were comparable to those in the overall study population. These findings may provide guidance for patients and clinicians in treatment decisions while also addressing other aspects of personalized medicine. Further studies on the oncologic outcomes of ICI-based combination therapies based on IMDC risk would help enrich our findings.

### Supplementary Information

Below is the link to the electronic supplementary material.

Supplementary file1 (DOCX 1305 kb)

Supplementary file2 (DOCX 166 kb)
