# Supplementary material for: Updated systematic review and network meta-analysis of first-line treatments for metastatic renal cell carcinoma with extended follow-up data
Source: Cancer Immunol Immunother. 2024 Jan 30;73(2):38. doi: 10.1007/s00262-023-03621-1 (PMC10827892; doi:10.1007/s00262-023-03621-1)

**Supplementary Figure 7** The referred Reporting Items for Systematic Reviews and Meta-Analyses (PRISMA) flow chart, detailing the article selection process


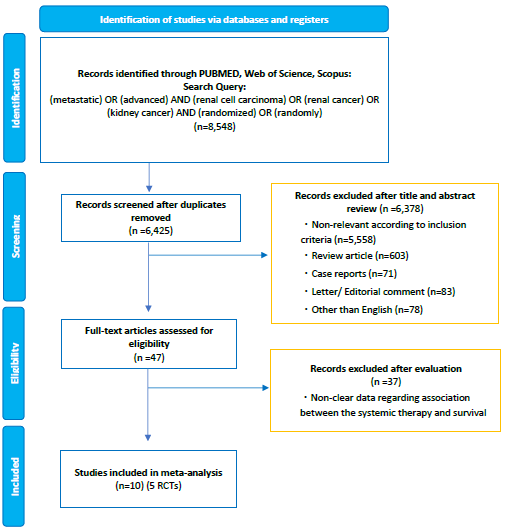


**Supplementary Figure 8** Forest plots showing the results of NMA for OS, PFS, ORR, and CR rate in mRCC patients with favorable risk treated with first-line ICI-based combination therapy


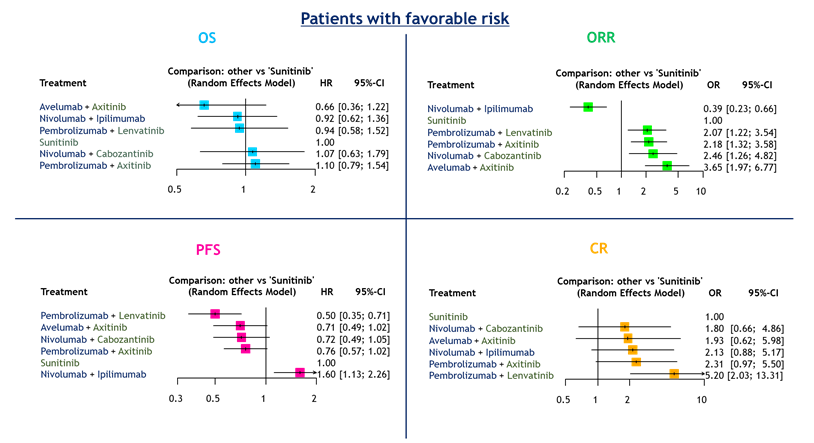

Supplement: Supplementary file 2 — Supplementary file2 (DOCX 166 kb) [file 262_2023_3621_MOESM2_ESM.docx]
